# SNPs and Somatic Mutation on Long Non-Coding RNA: New Frontier in the Cancer Studies?

**DOI:** 10.3390/ht7040034

**Published:** 2018-11-16

**Authors:** Linda Minotti, Chiara Agnoletto, Federica Baldassari, Fabio Corrà, Stefano Volinia

**Affiliations:** LTTA, Department of Morphology, Surgery and Experimental Medicine, University of Ferrara, Via Fossato di Mortara 70, 44123 Ferrara, Italy; mntlnd@unife.it (L.M.); chiara.agnoletto@unife.it (C.A.); bldfrc1@unife.it (F.B.); corra.fabio@unife.it (F.C.)

**Keywords:** lncRNAs, SNPs, somatic mutation, cancer risk

## Abstract

In the last decade, it has been demonstrated that long non-coding RNAs (lncRNAs) are involved in cancer development. The great majority of studies on lncRNAs report alterations, principally on their expression profiles, in several tumor types with respect to the normal tissues of origin. Conversely, since lncRNAs constitute a relatively novel class of RNAs compared to protein-coding transcripts (mRNAs), the landscape of their mutations and variations has not yet been extensively studied. However, in recent years an ever-increasing number of articles have described mutations of lncRNAs. Single-nucleotide polymorphisms (SNPs) that occur within the lncRNA transcripts can affect the structure and function of these RNA molecules, while the presence of a SNP in the promoter region of a lncRNA could alter its expression level. Also, somatic mutations that occur within lncRNAs have been shown to exert important effects in cancer and preliminary data are promising. Overall, the evidence suggests that SNPs and somatic mutation on lncRNAs may play a role in the pathogenesis of cancer, and indicates strong potential for further development of lncRNAs as biomarkers.

## 1. Introduction

### 1.1. Impact of SNPs on lncRNAs

Since 2010, genome-wide association studies (GWAS) have identified a large panel of single nucleotide polymorphisms (SNPs) across the human genome, that are associated with cancer susceptibility, prognosis and drug response [1]. SNPs represent the most common type of genetic variation within the population, with an incidence of one in every 100–300 nucleotides; about 10 million SNPs have been documented in the human genome [2]. Thus, SNPs hold great potential as markers for predictive analysis of cancer risk, prognosis, clinical outcome, drug resistance, and susceptibility to environmental factors [3]. Furthermore, in the last decade, more than 6500 disease-predisposing SNPs have been identified in over 1200 GWAS studies, although only 7% are located in protein-coding regions [4]. In contrast to the earlier candidate gene-driven approaches, GWAS constitutes a somewhat unbiased technology in identifying disease-associated loci [5]. SNPs and somatic mutation could disrupt the RNA secondary structure of the lncRNAS, affecting their molecular function and having an effect on their expression pattern [6]. In 2014, Bhartiya et al. found that the distributions of the variation in long non-coding RNAs (lncRNAs) present distinct patterns in potential functional elements [7].

### 1.2. LncRNAs Involved in Cancer

LncRNAs are emerging as a major class of non-coding transcripts. They are defined as non-protein-coding RNAs, more than 200 nt in length, and lacking an open reading frame of significant length (less than 100 nucleotides). The majority of lncRNAs appear indistinguishable from mRNAs, since they present 5′ cap structures and 3′ poly(A) tails; however, lncRNAs without a poly(A) tail have also been described [8]. Due to the recent discovery of a great number of lncRNAs and their diverse functions and complexity, this class of ncRNAs is classified by several criteria, including the genomic localization, modes of action, and function. Thus, depending on their genomic position, lncRNAs are subdivided into five categories: (i) sense-overlapping, if the lncRNA overlaps with one or more introns and exons of a protein-coding gene and is transcribed in the same direction; (ii) antisense, if the lncRNA originates from the antisense strand, complementary to protein-coding sequences; (iii) intronic, if the lncRNA originates from an intron of another protein-coding transcript; (iv) bidirectional, if the lncRNA is positioned in close vicinity to another coding transcript situated on the opposite strand; and (v), intergenic, if the lncRNA constitutes an independent unit within the sequence between two protein-coding regions [9,10]. Functionally, lncRNAs are classified based on their molecular mechanism of action; thus, signaling, decoy, guide, and scaffold lncRNAs have been identified as summarized in a recent reviews [11]: (1) signal lncRNAs activate or silence other transcripts. Their expression is cell type-specific and under tight transcriptional control to respond to diverse stimuli, thus, they act as markers of functionally significant biological events; (2) decoy lncRNAs compete with transcription factors and RNA-binding proteins, or miRNA, for their interactions with specific targets; (3) guide lncRNAs recruit selected chromatin-modifying enzymes to their specific target sequence, either in cis or in trans; (4) scaffold lncRNAs bind multiple proteins together to keep them closer to ribonucleoprotein complexes, and stabilize nuclear structures or signaling complexes, or facilitate their action on histones [11].

The most recent research from the ENCODE project reports that the human genome encodes more than 28,000 distinct lncRNAs, the majority of which are yet to be annotated [10]. By analyzing the transcriptome profiles of several cancer types using next-generation sequencing, the aberrant expression and the presence of mutations in a great number of lncRNAs has been documented [10]. Indeed, alterations in the expression of lncRNAs and their mutations promote tumorigenesis and metastasis. LncRNAs can function both as oncogenes or tumor-suppressors, regulating proliferation, survival, invasion, metastasis and angiogenesis of cancer cells. Although lncRNAs can be up- or down-regulated in cancer, the majority are up-regulated with respect to their canonical expression in normal tissues [10]. Recently, it has been confirmed that lncRNAs can modulate several pathways relevant for cancer development by interacting with other cellular components such as DNA, protein, and RNA [12]. Recent advancements in understanding the molecular mechanisms of action in lncRNAs are now providing the tools to functionally annotate these cancer-associated transcripts, and are revealing great potential for further application as targets for therapeutic intervention or as markers to predict tumor risk and prognosis for clinical applications [12].

## 2. Methods for Detection of SNPs and Measurement of Association with Cancer Risk

**Figure 1:**
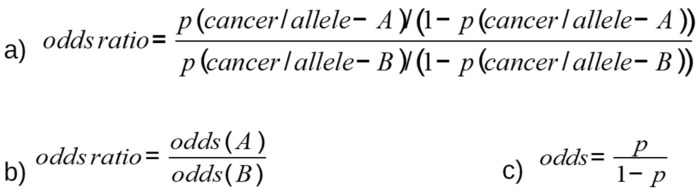
OR equation describing the association of the presence of SNP with the risk of tumor development.

SNPs are genotyped by analyzing groups of individuals and studying the association of SNPs with specific traits [13]. By measuring allele frequency between patients with tumors and healthy individuals, the role of SNPs relative to disorders can be determined. To genotype SNPs, the competitive allele-specific PCR assay, combined with a novel, fluorescence-based reporting system, is frequently used. Using this technology, any genetic variation occurring at the nucleotide level can be identified and measured, thus detecting SNPs or insertions and deletions [14]. The association rates are calculated through multivariate logistic regression analysis, which provides the odds ratio (OR) and related confidence intervals (95% CIs). The OR is defined as the measure of association derived from case-control studies, and is calculated as the ratio of two odds (Figure 1): first, the probability of the allele presenting with a specific SNP, and consequently developing a tumor (Figure 1a); and second, the probability of a cancer disorder presenting if the alternative allele is present (Figure 1b). The odds (Figure 1c) are defined as the probability of developing cancer relative to the probability of not developing cancer. An OR value >1 means that allele A effectively constitutes a risk factor for the development of cancer. Alternatively, an OR value <1 indicates that allele A exerts a protective function against tumors.

Polymorphisms in human genes have been described in remarkable numbers. Association studies to determine which polymorphisms are associated with cancer need to be designed according to strict protocols to obtain reliable results [15]. A genetic association case-control study compares the frequency of alleles or genotypes at genetic marker loci, usually SNPs, to determine whether a statistical association exists between the disease trait and the genetic marker. Recently, protocols have been written extensively describing the correct and efficacious planning of case-control studies, and providing tools and novel approaches for the efficient analysis and selection of markers [16]. Furthermore, protocols include methods of performing data quality control and basic statistical analysis to address the challenges in detecting and characterizing interactions among multiple factors, and to estimate the classification and prediction error of multifactorial models [17]. The success of a genetic association study depends on each of these steps.

**Figure 2:**
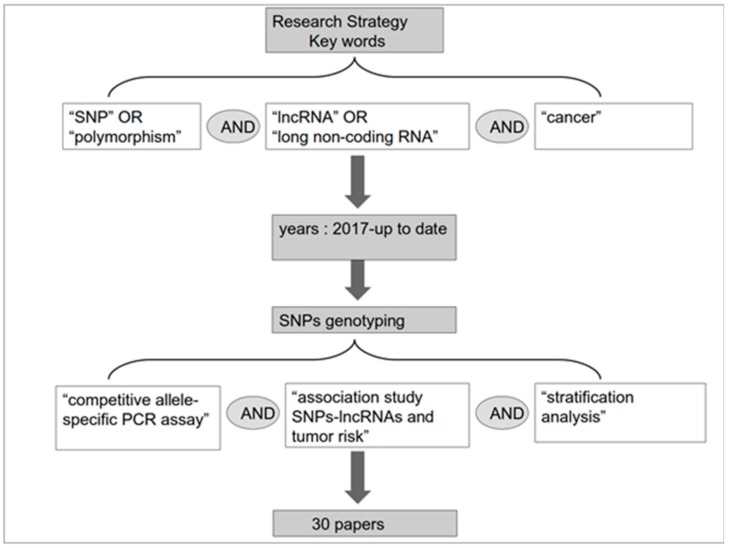
PRISMA diagram with details on the selection of articles for the review.

In this review, we summarize the current literature on the lncRNA-SNP axis in cancer from 2017 to the present as shown in the PRISMA diagram in Figure 2. The search strategy used in PubMed included the following Boolean query: (“lncRNA” or “long non-coding RNA”) AND (“polymorphism” or “SNP”) AND (“cancer”). Additional selection of articles was based on the method used for detection of SNPs and successive investigative stages; i.e., an in-depth competitive allele-specific PCR assay, association study of SNPs/lncRNAs and tumor risk, and stratification. The results of these articles are reported in Table 1. 

Table 1 presents a summary of the studies in PubMed published between January 2017 and August 2018, describing the most relevant SNPs in lncRNAs associated with cancer. In all reported studies, the analysis of polymorphisms, genotyping and stratification analysis were performed. The tumor type, model of cancer risk, subgroups of patients at risk, association with environmental factors, and if available, the PMID of the papers and the size of the cancer patient cohort and normal control cohort tested are reported for each SNP for all the lncRNAs in the list. 

### 2.1. SNPs on LncRNAs Involved in Cancer

**Hepatocellular carcinoma** (HCC) is one of the most frequently diagnosed cancers, and the most common type of primary liver cancer, and originates from chronic liver injury [18]. SNPs in coding genes and in non-coding genes have been reported to be associated with HCC risk [19], suggesting a potential function of SNPs in HCC prediction. However, few studies analyzed polymorphisms in lncRNA as a predictive biomarker for HCC. The highly up-regulated in liver cancer (HULC) lncRNA, firstly discovered in 2007, was identified as the mostly up-regulated ncRNA in HCC, and acts as an oncogene in multiple tumors [20]. HULC exerts multiple functions: it promotes tumor cell proliferation and induces autophagy and chemoresistance in HCC. Recently, it has been confirmed that the rs1041279 SNP in the promoter region of HULC could enhance HCC risk without significantly altering the expression level of the ncRNA. On the other hand, a MDR (multifactor dimensionality reduction) [21] analysis showed that the interaction of the rs2038540 SNP, detected in the intronic region of the lncRNA, with environmental factors, such as smoking and alcohol consumption, increases the risk of HCC [22]. A gene association study was recently performed with twelve potentially functional tag single nucleotide polymorphisms (tagSNPs) covering three onco-lncRNA genes, namely HOXA transcript at the distal tip (HOTTIP), colon cancer-associated transcript2 (CCAT2) and metastasis-associated lung adenocarcinoma transcript 1 (MALAT1), to investigate their association with HCC risk and prognosis. Selected SNPs were identified as potential predictive biomarkers. In particular, three SNPs (rs17501292, rs2067087, and rs17427960) in HOTTIP were associated with the risk of cancer under allelic models. The rs4102217 SNP in the promoter region of MALAT1 increased the risk in the dominant model, and the rs3807598 haplotype in HOTTIP intensified the risk of developing a tumor in females under 60 years of age. In addition, the rs3807598 variant in HOTTIP showed significantly longer survival time in the hepatitis B virus negative (HBV-negative) patients, while the rs591291 SNP in MALAT1 was associated with a significantly better prognosis in females and the HBV- negative subgroup [23]. eQTL (expression quantitative trait locus) analysis, undertaken to investigate the effects on lncRNA expression of the SNPs associated with HCC risk, revealed that the heterozygote genotype of intronic rs17427960 of HOTTIP was associated with higher expression [23]. Another independent potential marker predictor for HCC risk and prognosis is the SNP rs2839698 on lncRNA-H19 gene (H19). Furthermore, this SNP was associated with a poor prognosis in the smoking subgroup of a stratified analysis, thus functioning as a marker for environmental susceptibility. Another mutation in H19, the rs3024270 SNP, significantly increased the risk of HCC only in the <60 years subgroup [24].

**Prostate cancer** (PCa) is the second most common malignancy diagnosed in men, and is one of the major causes of cancer-related morbidity and mortality [25]. Thus, studies on cancer-associated risk have great importance for the prognosis of this aggressive and heterogeneous disorder. Recently, the Hox transcript antisense intergenic RNA (HOTAIR) lncRNA has been associated with cancer patients’ overall survival, metastatic potential, tumor recurrence and chemotherapy response, and the relevance of HOTAIR genetic polymorphisms in cancer risk has been established in several cancer types [26]. In particular, the homozygous CC genotype in the rs12826786 SNP is significantly associated with a subset of tumors, in particular with shorter recurrence-free survival in patients harboring locally advanced tumors (pT3-stage) [27]. In a recent meta-analysis, the effects of lncRNA RNA Polymerase II Subunit E (POLR2E) SNPs were analyzed in PCa, and preliminary data indicated that the rs3787016 SNP might increase the susceptibility to cancer in all the genotype models [28]. The rs11672691 SNP, which is associated with PCa risk, maps to the promoter of a short isoform of lncRNA Prostate Cancer Associated Transcript 19 (PCAT19-short), which is positioned within an intron of the long isoform (PCAT19-long). Of note, this SNP is bifunctional and mediates a switching between promoter and enhancer activity. Respectively reduces and increases the levels of PCAT19-short and PCAT19-long, thus causing both initiation and progression of PCa [29]. In this study, Hua et al. demonstrated the functional mechanisms of rs11672691 in PCa progression through the up-regulation of a lncRNA isoform, PCAT19-long. In particular, the PCa risk-associated variants at rs11672691 and rs887391 decrease NKX3.1, a well-known transcription factor frequently lost in PCa, and YY1 binding to the promoter region of PCAT19-short, altering this region to an enhancer for PCAT19-long [29]. 

The lncRNA prostate cancer associated non-coding RNA 1 (PRNCR1) is often up-regulated in PCa and modulates androgen receptor activity. Moreover, rs13252298, rs1456315 and rs7841060 polymorphisms in PRNCR1 were found to increase the risk of tumors in an Iranian population [30]. Again, a recent study in an Iranian population identified the SNPs rs4977574, rs1333048 and rs10757278 on the lncRNA Noncoding Antisense RNA in the INK4 Locus (ANRIL) as being associated with PCa risk and benign hyperplasia, suggesting a key role in the pathogenesis of both disorders [31]. The ANRIL-rs10757278 risk allele (G allele) has been shown to interfere with a binding site for STAT1, an IFN-γactivated transcription factor [32].

**Lung cancer**, including non-small-cell lung cancer (NSCLC) and small-cell lung cancer (SCLC), is a major cause of cancer-related death and has a very poor overall survival rate [33]. So far, there are a great number of association studies lncRNA/SNPs that confirming a link between these and tumorigenesis risk. Furthermore, exist a link between lncRNA/SNPs and chemotherapy response in cancers. HOTTIP rs1859168 or rs5883064, H19 rs2107425, and CCAT2 rs6983267 had a strong association with risk of lung cancer, while MALAT1 rs619586, H19 rs2107425 or rs2839698, CCAT2 rs6983267, HOTAIR rs1899663, or rs7958904 and ANRIL rs10120688 or rs1333049 were associated with platinum-based chemotherapy response in lung cancer patients [34]. Also, the rs10505477 SNP in lncRNA Cancer susceptibility candidate 8 (CASC8) could represent both a marker for response and toxicity to platinum-based treatment and for the risk of lung cancer [35]. Conversely, there were no statistically significant associations between rs4848320 and rs1110839 in AC016683.6 lncRNA and risk of lung cancer. However, by performing a stratification analysis based on environmental risks, both polymorphisms significantly increased the risk of lung cancer in dominant and homozygous models of smoking exposure [36]. LncRNA maternal expressed-gene 3 (MEG3) rs4081134 SNP was significantly associated with lung cancer susceptibility in a dominant model in the Chinese population [37]. The authors speculated that rs7158663 and rs4081134 may change the expression levels of MEG3 and affect the risk of lung cancer, but they omitted to observe the transcription factor binding sites involved in cancer development. By using the RNAfold web server, they also predicted that rs4081134 polymorphisms might alter the centroid secondary structure and minimal free energy, and thus change the folding of MEG3. However, this speculation requires further investigation [37]. A separate study in the Chinese population suggested that AC008392.1 rs7248320 may be involved in genetic susceptibility to NSCLC, in the population over 60 years of age. Conversely, the gene-environment interaction estimated on an additive scale had no significant results [38]. Several reports documented how over-expression of HOTAIR is associated with tumorigenesis and multiple cancer types, including lung cancer. The association between two polymorphisms, rs12826786 and rs1899663, and the risk of lung cancer was assessed in a Turkish population, and the analysis confirmed that carriers of Trs12826786/Crs1899663 present an increased risk of lung cancer susceptibility [39]. 

**Neuroblastoma** is the most frequently diagnosed extra-cranial cancer in children, after leukemias and cancer of the central nervous system [40]. Polymorphisms in MEG3 have been studied, but no susceptibility found to be conferred by either polymorphism, alone or in combination. Nevertheless, by stratification analysis, subjects carrying both rs4081134 and rs7158663 genotypes, show a higher risk of developing neuroblastoma than those with 0–1 risk genotype This was detected among the subgroup older than 18 month of age and in the group with clinical stage III+IV disease, thus suggesting MEG3 as a weak-effect neuroblastoma susceptibility gene [41]. Recently, the correlation between neuroblastoma susceptibility and HOTAIR has been reported in Chinese children. By association analysis and further stratification, three HOTAIR SNPs, rs12826786, rs874945 and rs1899663, were identified as being associated with increased neuroblastoma risk, predominantly in females and among patients with a tumor in the retroperitoneal region or mediastinum [42]. Also, in a Chinese case–control study, the association of the GWAS-identified lncRNA LINC00673 rs11655237 polymorphism with neuroblastoma susceptibility was assessed, confirming that this allele significantly increased the risk of tumor, particularly in cancer originating from the adrenal gland and clinical stage IV neuroblastoma [43].

**Medulloblastoma**, which is defined as an embryonal tumor of the cerebellum, is the single most common form of malignant brain tumor of childhood, and constitutes 20% of all primary central nervous system tumors [44]. The germ line lncRNA Cyclin Dependent Kinase Inhibitor 2B antisense (CDKN2BAS) SNP rs2157719, identified by GWAS, was genotyped in a Chinese population, and confirmed to be significantly associated with an increased medulloblastoma risk in a dominant model. The stratification analysis revealed that the predisposition of the polymorphism to medulloblastoma is higher in males [45].

**Colorectal cancer (CRC)** is the development of a tumor in the large part of the intestine, namely the colon and rectum [46]. Three association studies have been conducted on the same cohort of CRC and healthy samples. The first paper found a negative association between the risk of CRC and lncRNA RP11-3N2.1 with rs13230517 polymorphism [47]. The second study reported a protective effect of rs1194338 SNP on MALAT1, which is associated with decreased MALAT1 expression in CRC samples compared with normal controls [48]. The third study focused on the promoter region of lncRNA RP11-392P7.6 which is an antisense to the G-protein coupled receptor family C group 5-member D (CPRC5D) a coding region of the cancer-related gene. The SNP rs10845671 on lncRNA RP11-392P7.6 is associated with an increased risk of CRC, indicating that it may contribute to the CRC susceptibility and may be a candidate biomarker for CRC risk prediction [49]. LncRNA colorectal cancer associated transcript 1 (CCAT1) is a relatively novel lncRNA, whose over-expression is demonstrated in early stage of tumorigenesis and in later disease phases of CRC. Furthermore, an increase of CRC risk has been associated with two CCAT1 SNPs, rs7013433 and rs67085638, in different clinical stages [50].

Tissue differentiation-inducing non-protein-coding RNA (TINCR) is a key lncRNA required for somatic tissue differentiation and tumor progression, that is involved in the risk and progression of CRC. There are two SNPs on it, rs2288947 and rs8105637, that have been found to be associated with the decrease or increase of cancer risk respectively, validating its key role in the development of CRC [51].

In **bladder cancer** [52], the SNP rs874945 on HOTAIR has been associated with tumor risk in a hospital-based case-control study on more than two thousand samples. In the stratification analysis, this association was found to be more evident in the never-smoking group older than 60 years [53].

**Breast cancer** (BC) is the most common cancer and the second leading cause of cancer-related mortality in women worldwide. Both genetic risk and environmental factors contribute to its occurrence [54]. It has been demonstrated that HOTAIR increases BC risk and, in an association study on a female BC patient, it was found that, compared with healthy tissue, three SNPs were associated with cancer risk. Respectively, rs920778 and rs12826786 increased the BC risk while a negative correlation was found for rs1899663 SNP [55].

H19-rs217727 SNP was associated with susceptibility for **oral squamous cell carcinoma (OSCC)** and **osteosarcoma** risk with different alleles. OSCC is a malignant epithelial neoplasm affecting the oral cavity and osteosarcoma is a bone malignancy which occurs primarily in adolescents [56,57]. These findings indicate that H19-rs217727 SNP may play a role in genetic susceptibility to cancer pathogenesis risk [58,59].

A study of **pancreatic cancer** [60] and paired normal tissue found that SNP rs1859168 decreased pancreatic cancer risk deregulating HOTTIP expression [61]. rs1859168, was previously proposed to affect transcription factor binding sites and later the centroid secondary structure with a consequently minimum free energy, which might influence HOTTIP expression and function [34].

Recently, the effects of **gastric cancer** [62] associated lncRNAs on the susceptibility to this tumor have been assessed in a central Chinese population, confirming the protective role of lncRNAs SNPs (lnc-AMFR-1:1-rs4784659, lnc-ZNF33B-2:1-rs579501 and lnc-EVX1-3:3-rs1859168), thus providing potential biomarkers for the early diagnosis and therapy for tumors [63]. In the same study, bioinformatic analysis predicted that the genetic variation of lnc-AMFR-1:1-rs4784659 and lnc-ZNF33B-2:1-rs579501 leads to the change of the secondary structure, which may affect the binding capacity of miRNA and regulate the expression of lncRNA. However, the biological functions of lnc-AMFR-1:1 and lnc-ZNF33B-2:1 are not clear [63].

### 2.2. Somatic Mutation of lncRNAs in Cancer

Owing to recent developments in machine-learning technologies, next generation sequencing of pan-cancer data provides vast resources that aid the discrimination of somatic variants from germline variants, within non-coding regions. Thus, non-coding somatic mutations can be differentiated from background genomic regions, and associated features, such as copy number variations, conservation, substitution types and histone marker features, can be assessed [64]. To date, only a few articles are available in the literature on “somatic mutation”, lncRNA and cancer. A recent study has analyzed an extended list of ncRNAs with somatic copy number alteration (SCNA), as defined by the MiTranscriptome project, in order to identify either cancer-associated mutations or mutations in conserved regions [65]. In parallel, Singh et al. developed the mutation identification for RNA alterations (MIRA) method to study significantly mutated regions (SMR) affecting binding sites for RNA-binding proteins (RBP) in cancer. They found one exon SMR and 11 intron SMRs in TCL6, suggesting that lncRNA introns could be as relevant as exonic sequences in tumorigenesis [66]. Moreover, a study on papillary thyroid carcinoma (PTC) has shown that the lncRNA GAS8 Antisense RNA 1 (GAS8) is the second most frequently altered gene in this tumor type, confirming its oncosuppressor role [67]. Mularoni et al. employed the OncodriveFML method to analyze the pattern of somatic mutations across tumors in both coding and non-coding genomic regions in order to identify signals of positive selection involved in tumorigenesis. Briefly, MALAT1, a lncRNA gene previously shown to be involved in tumorigenesis of lung adenocarcinomas, and MIAT, a non-protein-coding transcript associated with myocardial infarction, presented a higher number than expected of function-impacting somatic mutations, thus assessing the reliability of this method [68]. Finally, a whole-genome landscape of somatic alterations performed in 300 Japanese individuals with liver cancer has shown recurrently mutated lncRNA genes, such as NEAT1 and MALAT1 [69]. Finally, a comprehensive evaluation of the genomic landscape in 452 chronic lymphocytic leukemia (CLL) cases identified novel recurrent mutations in non-coding regions, but not in lncRNA genes [70]. 

## 3. Discussion and Conclusions

Most of the tumor types we briefly discussed in the paragraphs above lack effective therapies and methods for early screening and stratification of patients, amenable for a precision medicine approach. Thus, implementing the panel of SNPs confirmed for any association with tumor risk, even in selected subgroups of patients, could certainly help to develop new strategies for clinical applications. Although preliminary and still incomplete, the set of data on the association of polymorphism in lncRNAs with cancer susceptibility might suggest that SNPs in lncRNAs hold great potential as prognostic biomarkers for cancer. Due to the novelty of the projects and studies on these recently discovered non-coding transcripts, much of the data on lncRNA mutations associated with cancer need, of course, to be further validated in prospective studies on larger sets of cohorts of patients, and for different ethnicities to obtain higher reliability. It is in fact possible that many of the reported observations might not be cross-validated or might not be generalizable to different populations worldwide. Moreover, the risks attached to SNPs describe only a minor component of the overall risk in cancer. Nonetheless, these genetic studies might be some of the very few that can implicate the otherwise elusive lncRNAs in the molecular pathways of cancer. It is indeed very difficult to assign a function to a lncRNA; for example as that of a regulator of RNA interactions or that of a scaffold and a protein binder.

The great majority of experimental data on variant genotypes, collected to date, consist principally of association studies, with a significant number evidencing only a tendency for altered expression or function of lncRNAs in cancers due to SNPs or other somatic mutations. Moreover, they require an inclusive analysis of public databases to confirm the preliminary observations in patients or to provide supporting evidence of a SNP role in other tissues, indicating that they could be functional. On the other hand, public databases often report incomplete information on lncRNAs, due to both the still limited repositories available today, and the lack of effective and complete annotation of lncRNAs in the human genome.

Overall, there is still little experimental data that clearly demonstrates the cancer relevance of SNPs and somatic alterations in lncRNA. The most frequently mutated lncRNAs in cancers with a large impact in healthcare were undoubtedly HOTAIR, HOTTIP, and MALAT1, whose SNPs were detected in several studies, confirming their involvement in relevant cancer functions. Otherwise, SNPs in other lncRNAs were specific to selected cancer types, indicating they could operate in specific signaling mechanisms. The substantial knowledge gap that still exists between the SNP association and the molecular mechanism contributing to disease risk represents both a question and an opportunity in the identification of lncRNAs with key roles in cancer. A polymorphism in a regulatory element might alter the abundance of a gene transcript. However, a great number of SNPs are positioned in intergenic or intronic regions, and the relationship of these with lncRNA function in the cell context might be much more circumspect. To analyze the action of SNPs in this respect, expression quantitative trait locus (eQTL) mapping can be applied. With eQTL the relation of RNA expression with genotype data is measured to determine if a variant is correlated with gene transcription [71].

In parallel, bioinformatic approaches have frequently been used to predict the potential effects of genetic variations in the structure of lncRNAs. Despite the secondary structure of many lncRNAs have been resolved by using the SHAPE-directed RNA chemistry prediction tool [72,73], the analysis of the alterations due to SNPs on the secondary structure of lncRNAs have not yet been extensively investigated. The atlas Lnc2Catlas compiles quantitative associations between lncRNAs and cancers, using three computational methods that assess secondary structure disruption, lncRNA-protein interactions, and co-expression networks [74]. Furthermore, the updated version of lncRNASNP2 provides comprehensive information on SNPs and mutations in lncRNAs, and uses RNAsnp to assess variant effects on lncRNA secondary structure and function [75]. Moreover, some research on selected SNPs has been completed. The rs2366152C SNP on HOTAIR, for example, affected the secondary structure, with loss of the binding site for miR-22, in herpes virus (HPV)16-related cervical cancer development [76]. A novel risk SNP rs114020893 was predicted to change the secondary structure in the lncRNA NEXN-AS1 at 1p31.1, and may contribute to lung cancer susceptibility [77]. 

Overall, no data are currently available on the biological functions of the SNPs we describe in this review. Also, studies on somatic mutations of lncRNAs and their impact on cancer are still at an early stage.

As of today the most relevant implications of lncRNAs SNP studies include the cancer risk assessment and susceptibility. However, due to the evolution of next-generation sequencing and the advent of single cell sequencing, it is likely that a host of discoveries on the involvement of lncRNAs in cancer are finally on the horizon. In the near future, clinical applications of SNP detection in lncRNAs might include early cancer diagnosis, risk prediction for relapse or progression, monitoring the effects of systemic therapies, and patient stratification. Eventually, these studies could also lead to the identification of novel targets for the development of innovative therapies. This research field remains one of the major challenges for the study of SNPs and other variants of lncRNAs involved in cancer. 

## Figures and Tables

**Table 1:** Long non-coding RNAs (LncRNAs): their mechanism of action and significance in cancer.

lncRNA	SNP	Allele (Major/Minor)	Cancer Risk/Model	Environmental Risk	Subgroup Risk	Cancer	PMID	n. Cases Test	n. Controls Tests
AC008392.1	rs7248320	A/G	↓ recessive		↓ recessive > 60 years	lung NSCLC	29881308	512	588
AC016683.6	rs4848320	C/G/T		↑ dominant smoking		lung	29997452	434	593
AC016683.6	rs111083	A/C/G/T		↑ homozygous smoking		lung	29997452	434	593
AMFR-1:1	rs4784659	C/T	↓ dominant and additive			gastric cancer	29305976	470	470
ANRIL	rs4977574	A/G/T	↑ allelic		↑ BPH	prostate	28621612	125	220
ANRIL	rs1333048	A/C	↑ allelic		↑ BPH	prostate	28621612	125	220
ANRIL	rs10757278	A/G	↑ allelic		↑ BPH	prostate	28621612	125	220
CASC8	rs10505477	C/T		↓ dominant platinum-based chemotherapy resistance	↑ recessive male and adenocarcinoma	lung	27249003	498	213
CCAT1	rs7013433	A/C/T			↑ dominant late clinical stage	colon rectal cancer	29666003	507	503
CCAT1	rs67085638	C/T	↑ dominant			colon rectal cancer	29666003	507	503
CDKN2BAS	rs2157719	T/C	↑ dominant		↑ pediatric and males	medulloblastoma	29314442	160	443
EVX1-3:3	rs1859168	A/C/G/T	↓ dominant and recessive			gastric cancer	29305976	470	470
H19	rs2839698	C/T/A	↑ dominant	↑ ever smoking	↑ <60 years	hepatocellular carcinoma	29511035	472	472
H19	rs3024270	C/G			↑ <60 years	hepatocellular carcinoma	29511035	472	472
H19	rs217727	C/T	↑ additive			oral squamous cell carcinoma	28975993	362	741
H19	rs217727	G/A	↑ dominant			osteosarcoma	28975992	193	393
HOTAIR	rs874945	G/A	↑ dominant		↑ >60 years never smoking	bladder	29673865	1050	1407
HOTAIR	rs920778	C/T	↑ dominant			breast	29022495	220	231
HOTAIR	rs12826786	C/T	↑ dominant			breast	29022495	220	231
HOTAIR	rs1899663	G/T	↓ dominant			breast	29022495	220	231
HOTAIR	rs12826786	C/T	↑ dominant		↑ female	infant neuroblastoma	29603181	393	812
HOTAIR	rs874945	C/T	↑ dominant		↑ female	infant neuroblastoma	29603181	393	812
HOTAIR	rs1899663	C/A	↑ dominant		↑ female	infant neuroblastoma	29603181	393	812
HOTAIR	rs12826786	C/T	↑ combination			lung	29974853	87	93
HOTAIR	rs1899663	G/T	↑ combination			lung	29974853	87	93
HOTAIR	rs12826786	C/T			↑ shorter biochemical recurrence-free survival in pT3-stage	prostate	29436234	151	180
HOTTIP	rs17501292	T/C/G	↑ allelic			hepatocellular carcinoma	29930469	521	817
HOTTIP	rs2067087	G/C	↑ allelic			hepatocellular carcinoma	29930469	521	817
HOTTIP	rs17427960	C/A	↑ allelic			hepatocellular carcinoma	29930469	521	817
HOTTIP	rs3807598	C/G			↓ HBV-negative	hepatocellular carcinoma	29930469	521	817
HOTTIP	rs1859168	A/C	↓ dominant			pancreatic	28818070	416	146
HULC	rs1041279	C/G	↑ recessive	n.d	↑ male	hepatocellular carcinoma	29803923	517	810
HULC	rs2038540	C/G	n.d	↑ smokers-drinkers	n.d	hepatocellular carcinoma	29803923	517	810
LINC00673	rs11655237	C/T	↑ dominant		↑ patients with tumor originating from the adrenal gland	infant neuroblastoma	29339420	393	812
MALAT1	rs1194338	C/A	↓ dominant	n.d	n.d	colon rectal cancer	29190941	320	319
MALAT1	rs4102217	G/C	↑ dominant			hepatocellular carcinoma	29930469	521	817
MALAT1	rs591291	C/T			↓ HBV-negative and female	hepatocellular carcinoma	29930469	521	817
MEG3	rs7158663	G/A	↑ combination			infant neuroblastoma	29615542	392	783
MEG3	rs4081134	G/A			↑ recessive > 18 month	infant neuroblastoma	29615542	392	783
MEG3	rs4081134	A/G	↑ dominant			lung	30113224	526	526
PCAT19	rs11672691	A/G	↑ bifunctional			prostate	30033362		
POLR2E	rs3787016	C/T	↑ all genotype			prostate	29922603	5	5
PRNCR1	rs13252298	A/G	↑ recessive			prostate	29285392	178	180
PRNCR1	rs1456315	G/A	↑ allelic			prostate	29285392	178	180
PRNCR1	rs7841060	T/G	↑ allelic			prostate	29285392	178	180
RP11-392P7.6	rs10845671	A/C/T	↑ dominant			colon rectal cancer	28612367	320	319
RP11-3N2.1	rs13230517	G/A	↓ dominant	↓ non-drinkers	n.d	colon rectal cancer	29167551	320	319
TINCR	rs2288947	A/G	↓ dominant			colon rectal cancer	28418933	1400	1400
TINCR	rs8105637	A/G	↑ dominant			colon rectal cancer	28418933	1400	1400
ZNF33B-2:1	rs579501	A/C	↓ dominant and additive			gastric cancer	29305976	470	470

Note: Simbol (↑) meaning an increase of the risk and (↓) a reduction of risk.
